# Trehalose Ameliorates Diabetic Cardiomyopathy: Role of the PK2/PKR Pathway

**DOI:** 10.1155/2021/6779559

**Published:** 2021-12-21

**Authors:** Yuning Liu, Shi Wu, Qian Zhao, Zhen Yang, Xiaojun Yan, Cairong Li, Wenliang Zha, Wei Yu

**Affiliations:** ^1^Xianning Medical College, Hubei University of Science and Technology, Xianning 437100, China; ^2^School of Pharmacy, Xianning Medical College, Hubei University of Science and Technology, Xianning 437100, China; ^3^Medicine Research Institute, Xianning Medical College, Hubei University of Science and Technology, Xianning 437100, China

## Abstract

Ample clinical case reports suggest a high incidence of cardiomyopathy in diabetes mellitus (DM). Recent evidence supports an essential role of trehalose (TLS) in cardiomyocyte survival signaling. Our previous study found that prokineticin2 (PK2) was involved in the process of diabetic cardiomyopathy (DCM). The present study examined the protective effects and mechanisms of TLS on DM-induced cardiomyocyte injury in mice and H9c2 cardiomyocytes. C57BL/6J mice were intraperitoneally injected with 50 mg·kg^−1^·d^−1^ streptozotocin for five consecutive days to establish an experimental diabetic model and then administered TLS (1 mg·g^−1^·d^−1^, i.p.) for two days every 4 weeks and given 2% TLS in drinking water for 24 weeks. Echocardiography, myocardial structure, apoptosis, pyroptosis, autophagy, and the PK2/PKR pathway were assessed. Cardiomyocytes exposed to high glucose (HG) were treated with TLS in the absence or presence of the PK2 antagonist PKRA7, and proteins involved in apoptosis, autophagy, and pyroptosis and the PK2/PKR pathways were evaluated using Western blot analysis. Diabetic mice demonstrated metabolic disorder, abnormal myocardial zymograms, and aberrant myocardial systolic and diastolic function, which were accompanied by pronounced apoptosis, pyroptosis, and dampened autophagy. TLS treatment relieved these effects. PK2 and receptor expressions were downregulated in diabetic mice, and TLS nullified this effect. PKRA7 eliminated the impact of TLS on cardiomyocytes. This evidence suggests that TLS rescues DM-induced myocardial function, pyroptosis, and apoptosis, likely via the PK2/PKR pathway.

## 1. Introduction

The prevalence of diabetes mellitus (DM) in adults exceeds 10% in China, and European and American countries have rates that are three times higher than that in China; DM has become a major disease threatening human health [1, 2]. Diabetic cardiomyopathy (DCM) occurs due to persistent abnormal blood glucose and lipid metabolism associated with DM and leads to myocardial fibrosis, ventricular remodeling, and cardiac systolic and diastolic dysfunction, which is one of the main causes of death in diabetic patients [3]. The pathogenesis of DCM is relatively complicated, and diabetic dyslipidemia [3], mitochondrial damage [4], oxidative stress [5], myocardial fibrosis [6], apoptosis [7], pyroptosis [8], and autophagy abnormalities [9] are involved in the occurrence and development of DCM. Although existing antihyperglycemic treatments alleviate the development of DCM, the results did not meet expectations. The high prevalence rate and poor prognosis of DCM remain problems for clinical medical staff [10, 11]. Therefore, studying the pathogenesis of DCM and finding a prevention and treatment strategy are extremely urgent needs.

Trehalose (TLS) is a nonreducing disaccharide composed of two D-glucose units connected by glycosidic bonds, and it is widely found in animal and plant microorganisms. TLS is a metabolite of cells that resists adverse environmental stress. TLS is extensively used in biomedicine, food, cosmetics, agriculture, and other fields [12]. TLS has antidrying, anticold, and antihigh temperature and nonspecific protective effects in organisms [13]. TLS has attracted much recent attention because of their diverse array of biological and pharmacological activities, including enhancing autophagy, regulating glucose homeostasis, and exerting anti-inflammatory and antiapoptotic effects [14]. Insulin resistance is a pathogenic factor of DCM, and TLS may rescue insulin resistance-induced abnormal cardiac contractions [15]. However, the effect of TLS on DCM remains unknown.

Prokineticin2 (PK2), also known as human Bv8, is statically expressed in a variety of tissues, including the brain, heart, and testicles. PK2 participates in biological processes such as angiogenesis, hematopoiesis, immune response, and circadian rhythm regulation via binding to two highly homologous G protein-coupled receptors, prokineticin receptor 1 (PKR1), and prokineticin receptor 2 (PKR2) [16, 17]. PK2 and PKR play critical roles in cardiac homeostasis under physiological and pathological conditions [18]. The expression of PK2 was inhibited in patients with heart failure, and the levels of PKR1 may be suppressed, which eventually damages the cardiac structure [19, 20]. PK2/PKR1 signal transduction promotes the formation of cross-capillary insulin channels and increases insulin sensitivity [21]. The results from our study revealed that metformin inhibited cardiomyocyte apoptosis by regulating PK2/PKR pathway and ultimately restored the cardiac homeostasis of DM [22]. Therefore, this research evaluated the effects and possible mechanisms of TLS on DM-induced cardiomyocyte apoptosis, pyroptosis, and changes in autophagy.

## 2. Methods

### 2.1. Experimental Animals

The Committee of Experimental Animals of the Hubei University of Science and Technology approved the experimental procedures, which followed the National Institutes of Health (NIH) Guide for the Care and Use of Laboratory Animals. Eighty SPF male C57BL/6J mice (22 ± 2 g, 5-6 weeks old) were obtained from Pengyue Experimental Animal Breeding Co., Ltd. and used in this study. The mice were housed at a temperature of 22 ± 2°C and a moisture content of 40% under a 12 h light/dark cycle with free access to food and water.

### 2.2. Induction of Experimental DM Mice

The mice were adapted for one week before glucose challenge. Mice in the DM and DM-TLS groups were given intraperitoneal injections of a streptozotocin (STZ, Sigma, Germany) solution (50 mg·kg^−1^·d^−1^) in sodium citrate buffer (pH 4.5) for 5 consecutive days after a 12 h of fasting, and mice in the control and control-TLS groups were injected with the same volume of sodium citrate buffer. After 7 days of intraperitoneal injection, random blood glucose was determined via tail vein blood sampling, and blood glucose levels ≥ 16.7 mmol · L^−1^ were considered a diabetic mouse model. Unmodeled mice were discarded. Sixty mice were randomly divided into a normal control group (control group, *n* = 15), DM model group (DM group, *n* = 15), TLS control group (control-TLS group, *n* = 15), and TLS treatment group (DM-TLS group, *n* = 15). The control-TLS and DM-TLS groups received intraperitoneal injections of a TLS solution (1 mg·g^−1^·d^−1^) for two consecutive days every 4 weeks and 2% TLS in drinking water for 24 weeks. The control and DM groups were treated with an equal volume of saline.

### 2.3. Cell Culture and Treatment

Rat H9c2 cardiomyocytes (purchased from the China Center for Type Culture Collection) were cultured in DMEM low-sugar medium supplemented with 10% FBS and a 1% penicillin-streptomycin solution at 37°C and 5% CO_2_ in a humidified environment. Cells were incubated with 33 mM high glucose (HG) for 72 h with or without different concentrations of TLS (50, 100, and 150 mmol·L^−1^). PKRA7 was added to petri dishes to observe the effect of the PK2/PKR pathway.

### 2.4. MTT Assay

Succinate dehydrogenase in the mitochondria of living cells reduces MTT (3-(4,5-dimethylthiazol-2-yl)-2,5-diphenyltetrazolium bromide) to blue purple crystal formazan, and the quantity of methylpyrazine produced is directly proportional to the quantity of living cells. H9c2 cardiomyocytes were exposed to HG medium in the absence or presence of TLS for 72 h prior to assessments of cell viability. The MTT assay was performed according to the manufacturer's instructions.

### 2.5. Ad-GFP-LC3B Transfection

An adenovirus containing a GFP-LC3B construct was provided by Beyotime Biotechnology (Shanghai, China). Cells were transfected with GFP-LC3B adenovirus for 24 h and then refreshed with new medium. After 72 h, cells were visualized for autophagy using fluorescence microscopy and treated with normal or HG in the absence or presence of TLS or the autophagy agonist rapamycin.

### 2.6. General State Measurement

Blood glucose was measured at the end of 0, 4, 8, 12, 16, 20, and 24 weeks after TLS intervention. Glucose tolerance was assessed before the end of the experiment as previously described, with minor modifications. Briefly, mice were fasted for at least 12 h and intraperitoneally injected with a 2 g·kg^−1^ glucose solution [23]. Blood glucose was measured 0, 15, 30, 60, and 120 min after injection using a glucometer.

### 2.7. Echocardiographic Assessment

The cardiac structure and function of mice under anesthesia (1% isoflurane) were evaluated using M-mode echocardiography (Vevo®1100, VisualSonics, Toronto, Canada). Hemodynamic parameters were recorded from three consecutive cycles, such as the heart rate (HR), left ventricular ejection fraction (LVEF), left ventricular fractional shortening (LVFS), and the ratio of early to late left ventricular diastolic filling (*E*/*A* ratio).

### 2.8. Morphological Assessment

Hearts were removed and placed in 4% paraformaldehyde for 24 h before embedding in paraffin and being sectioned. The myocardial sections were stained with hematoxylin and eosin (HE) and Masson's trichrome and photographed under light microscopy at ×400 magnification. Approximately 1 mm^3^ of left ventricular tissue was fixed in 2.5% glutaraldehyde fixative for more than 2 h and postfixed with 1% osmium tetraoxide. These tissues were embedded in an acetone-812 embedding agent, double-stained with uranyl acetate and lead citrate, and cut into 60 nm thick sections. The specimens were imaged using transmission electron microscopy (HT7700, Hitachi, Tokyo, Japan).

### 2.9. Measurement of the Biochemical Index

Aspartate transaminase (AST), lactate dehydrogenase (LDH), creatine kinase (CK), and creatine kinase-MB (CK-MB) and the levels of total cholesterol (TC) and triglyceride (TG) in the serum were measured using an automatic biochemical analyzer (Olympus, Tokyo, Japan).

### 2.10. Terminal Deoxynucleotidyl Transferase-Mediated dUTP Nick End-Labelling (TUNEL) Assay

The apoptosis assay was performed using a TUNEL kit (Roche Applied Science, Indianapolis, USA) according to the manufacturer's instructions. After antigen repair, myocardial sections were incubated with TdT and dUTP, and images were captured using microscopy at ×400 magnification. The proportion of TUNEL-positive cells was estimated using the following formula: TUNEL positive cardiomyocytes/total number of cardiomyocytes ×100%.

### 2.11. Western Blot Analysis

Fifty milligrams of myocardial tissue was collected, and total protein was extracted from tissue lysates. The protein concentration was determined using the BCA method. Appropriate protein samples were separated using SDS-PAGE electrophoresis, and the proteins were transferred to PVDF membranes via electrical imprinting transfer. Antibodies against cleaved caspase-3, Bax, Bcl-2, light chain 3B (LC3B), Beclin-1 (1 : 1000, Cell Signaling Technology, USA), PK2 (1 : 1000, Abcam, USA), PKR1, PKR2 (1 : 2000, Santa Cruz Biotechnology, USA), GAPDH (1 : 10000, Proteintech, USA), ubiquitin-binding protein (p62) (1 : 500, Wanleibio, China), autophagy-related proteins (Atg5), NALP3, caspase-1, IL-18, and IL-1*β* (1 : 1000, Bioss, China) were incubated overnight at 4°C. Secondary antibodies were added and incubated at room temperature for 1 h. After ECL color development, Image Lab software was used to determine the band absorbance value. GAPDH expression was used as the loading control.

### 2.12. Statistical Analysis

The data are presented as the means ± SD of replicated experiments. Analysis was performed using t-test or one-way analysis of variance. Differences with *P* values < 0.05 were considered statistically significant.

## 3. Results

### 3.1. TLS Improved the General Features

**Figure 1:**
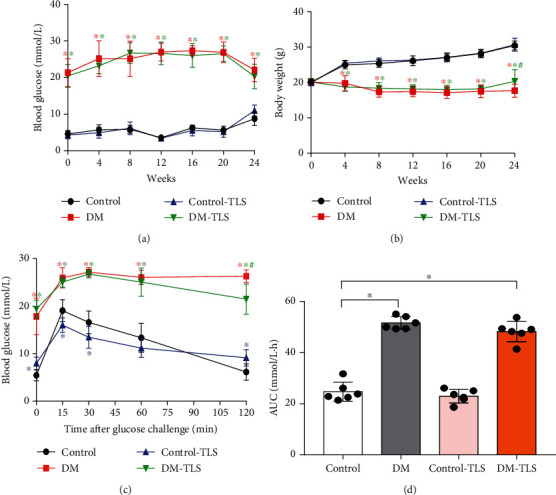
TLS effects blood glucose and body weight in diabetic mice. (a, b) Changes in blood glucose and weight in diabetic mice at different time points. *n* = 10 per group. (c, d) Effect of TLS on glucose tolerance in the abdominal cavity of diabetic mice. *n* = 6 per group. Means ± SD. ^∗^*P* < 0.05 compared to the control group, ^#^*P* < 0.05 compared to the DM group.

The body weight (BW) and glucose levels of mice were observed after glucose challenge. The levels of mice in each group were the same in the initial stage, but STZ caused sustained hyperglycemia and BW loss, which were significantly different than those in normal mice. At the end of 24 weeks, TLS improved these changes in diabetic mice, but it did not restore these changes to normal levels (Figures 1(a) and 1(b)).

Heart weight (HW) and lung weight (LW) intuitively reflect the level of cardiopulmonary function in mice and have an indicative effect on heart failure. Compared to the control group, the results showed that HW and LW were much lower, and the ratio of heart-to-body weight (HW/BW) and lung-to-body weight (LW/BW) was increased in the DM group. TLS partially reversed these changes (Table 1).

Abnormal glucose tolerance is a marker of insulin resistance and may be used as a flag to predict cardiovascular complications in DM [24]. Impaired glucose tolerance was observed in DM mice during intraperitoneal glucose tolerance tests (Figure 1(c)). Plasma glucose concentrations were increased at different times for 120 min after glucose injection in DM mice compared to control mice. Glucose tolerance was slightly improved in the DM-TLS group (Figure 1(c)). Taking the total area under the curve (AUC) for blood glucose as the quantitative result of the intraperitoneal glucose tolerance test, the AUC in the DM group had obvious increment compared with that in the control group, and TLS administration slightly reduced the AUC in DM mice (Figure 1(d)).

### 3.2. TLS Inhibits DM-Induced Cardiac Function and Structural Changes In Vivo

**Figure 2:**
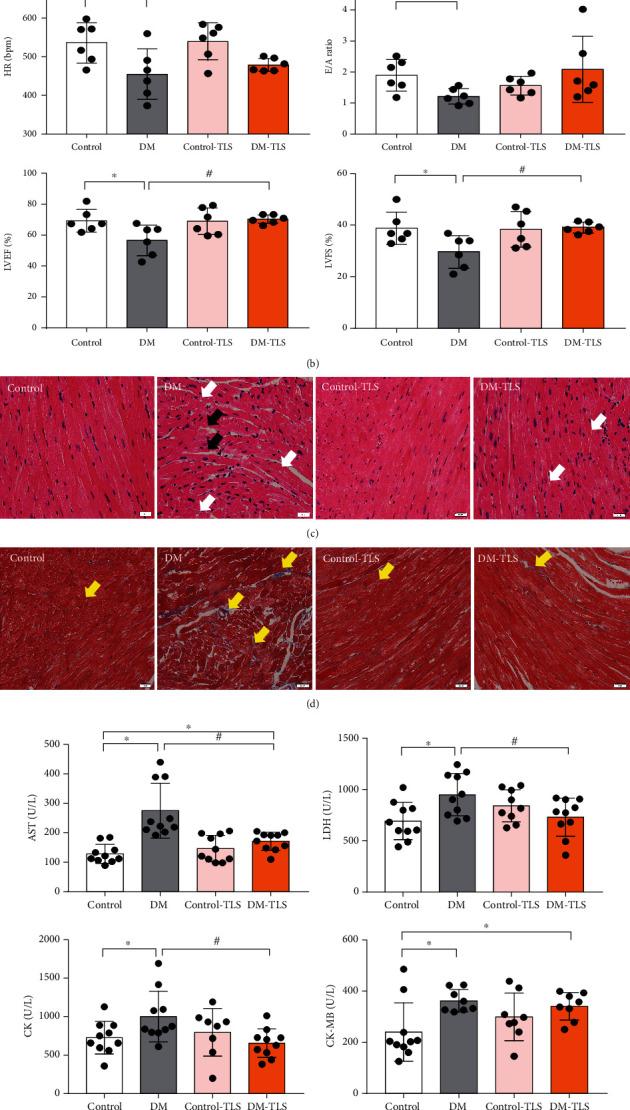
TLS changes the cardiac function and structure in diabetic mice. (a) Representative echocardiography image. (b) Quantification of echocardiography (*n* = 6 per group). (c) Representative image of HE staining (*n* = 5 per group). (d) Representative image of Masson trichromatic staining (*n* = 5 per group). (e) Quantification of serum myocardial zymograms. (f) Quantification of blood lipid. *n* = 8-10 per group. The black arrows indicate myocardial rupture, white arrows indicate myocardial vacuole, and yellow arrows indicate myocardial collagen deposition. Means ± SD. ^∗^*P* < 0.05 compared to the control group, ^#^*P* < 0.05 compared to the DM group.

Echocardiographic assessment revealed that DM caused a decrease in HR, LVEF, LVFS, and the *E*/*A* ratio. Although TLS failed to alter cardiac geometry and function in the control group, it partially eliminated DM-induced changes in echocardiographic indices (Figures 2(a) and 2(b)).

HE staining showed disordered myocardial arrangement, such as nuclear vacuolization, and a large myocardial space was observed in the DM group. TLS ameliorated these morphology changes in cardiac tissue (Figure 2(c)). To further verify the effect of TLS on cardiac fibrosis in mice, Masson trichromatic staining was performed. The results showed that perivascular collagen was meaningfully increased in the DM group, and the collagen content of TLS-treated mice was obviously lower than that of the DM group (Figure 2(d)).

Myocardial enzymes, including LDH, AST, CK, and CK-MB, are recognized markers of myocardial damage and necrosis and were significantly elevated in the DM group compared to the control group. TLS mitigated these alterations. TG and TC levels were remarkably increased in DM mice, and TLS treatment marginally reduced these effects (Figures 2(e) and 2(f)).

### 3.3. TLS Inhibits DM-Induced Apoptosis and Pyroptosis In Vivo

**Figure 3:**
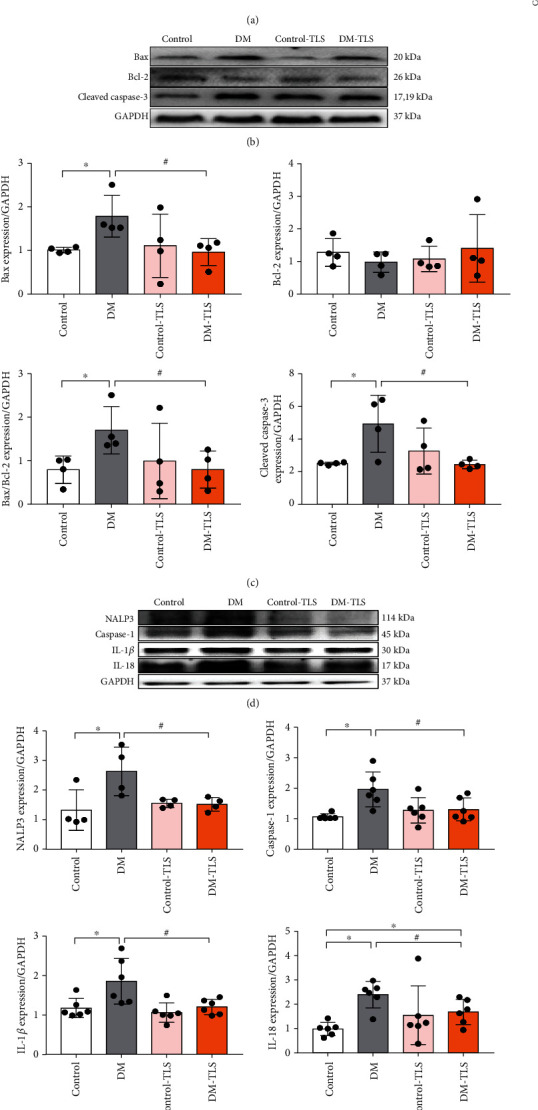
TLS reduces cardiac apoptosis and pyroptosis in diabetic mice. (a) Representative image of TUNEL staining and analysis of positive cells. *n* = 3 per group. (b) Representative protein expression of Bax, Bcl-2, and cleaved caspase-3. (c) Quantification of Bax, Bcl-2, and cleaved caspase-3 protein expression. (d) Representative protein expression of NALP3, caspase-1, IL-1*β*, and IL-18. (e) Quantification of NALP3, caspase-1, IL-1*β*, and IL-18 protein expression. *n* = 4-6 per group. The values are presented as the means ± SD. ^∗^*P* < 0.05 compared to the control group, ^#^*P* < 0.05 compared to the DM group.

To measure whether STZ-induced cardiac dysfunction occurred because of heart remodeling, we assessed cardiac apoptosis and pyroptosis as well as their biomarkers. DM group mice showed a higher number of apoptotic bodies compared to the control group, and the number of apoptotic cells decreased after TLS treatment (Figure 3(a)). The pathological apoptosis marker cleaved caspase-3 and the Bax-to-Bcl-2 ratio were increased in the DM group, and TLS administration markedly normalized these changes (Figures 3(b) and 3(c)). To demonstrate whether TLS influenced NALP3-mediated pyroptosis in STZ-induced mice, the expression of pyroptosis-related proteins was measured using Western blot. STZ-induced pyroptosis presented as an upregulation of NALP3, caspase-1, IL-18, and IL-1*β*, which were notably reversed by TLS. TLS itself exerted little effect on these pyroptosis markers (Figures 3(b) and 3(e)).

### 3.4. TLS Protects against DM-Induced Autophagy Reduction In Vivo

**Figure 4:**
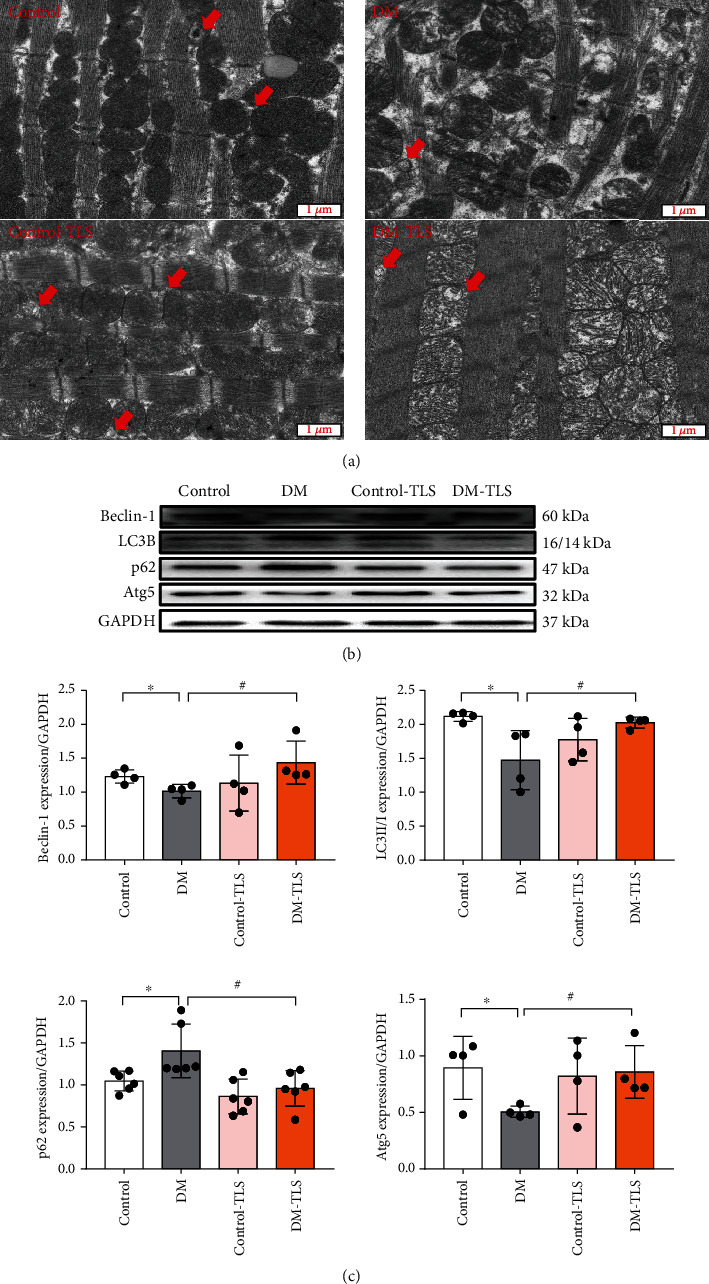
TLS activates autophagy in diabetic mice. (a) Representative transmission electron microscopy image. Red arrows mark autophagosomes. (b) Representative protein expression of Beclin-1, LC3B, p62, and Atg5. (c) Quantification of Beclin-1, LC3B, p62, and Atg5 protein expression. *n* = 4-6 per group. Means ± SD. ^∗^*P* < 0.05 compared to the control group, ^#^*P* < 0.05 compared to the DM group.

Autophagy plays a key role in cardiomyocyte survival. As shown in Figure 4, broken dissolved myocardial fibers, swollen mitochondria, and a decrease in autophagosomes were observed in DM mice using transmission electron microscopy, and TLS effectively rescued these changes induced by DM. Western blot analysis revealed that the levels of autophagy protein markers, including Beclin-1 and Atg5 expression, and the LC3II/I ratio were notably decreased, and p62 protein expression increased in the DM group. TLS treatment marginally negated these effects. TLS itself produced little effect.

### 3.5. TLS Improves the Expression of the PK2/PKR Signaling Pathway in DM Mice

**Figure 5:**
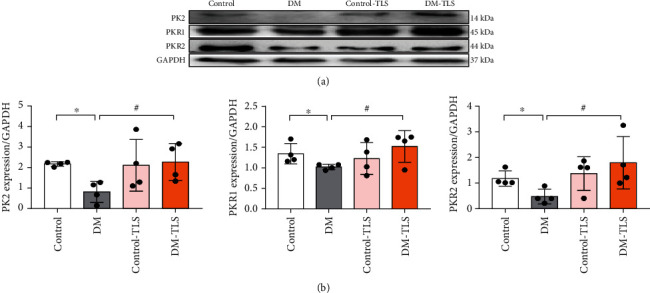
TLS activates the cardiac PK2/PKR pathway in diabetic mice. (a) Representative protein expression of PK2, PKR1, and PKR2. (b) Quantification of PK2, PKR1, and PKR2 protein expression. *n* = 4 per group. Means ± SD. ^∗^*P* < 0.05 compared to the control group, ^#^*P* < 0.05 compared to the DM group.

The protein expression levels of PK2, PKR1, and PKR2 in the myocardium of the DM group were markedly reduced compared with those of the control group (Figure 5(a)). After TLS treatment, the protein expression levels of PK2, PKR1, and PKR2 were increased to the level of the control group (Figure 5(b)). These data suggest that TLS had a beneficial effect on DM-evoked myocardial injury via amelioration of the PK2/PKR pathway.

### 3.6. TLS Suppresses HG-Triggered H9c2 Cardiomyocyte Injury

**Figure 6:**
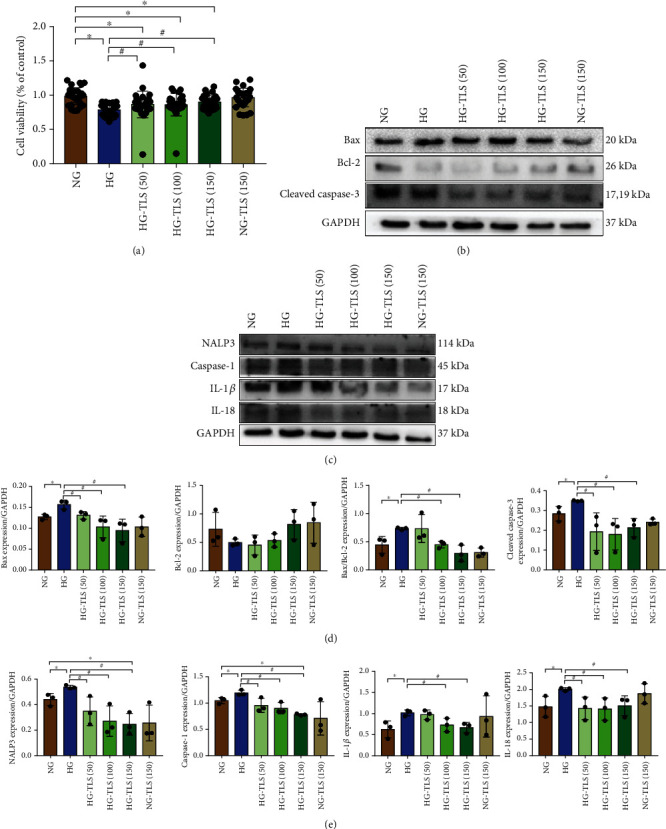
TLS suppresses HG-induced H9c2 cardiomyocyte injury. (a) Cell proliferation. (b) Representative protein expression of Bax, Bcl-2, and cleaved caspase-3. (c) Representative protein expression of NALP3, caspase-1, IL-1*β*, and IL-18. (d) Quantification of Bax, Bcl-2, and cleaved caspase-3 protein expression. (e) Quantification of NALP3, caspase-1, IL-1*β*, and IL-18 protein expression. GAPDH served as the loading control. Means ± SD, *n* = 3 cultures per group. ^∗^*P* < 0.05 compared to the NG group, ^#^*P* < 0.05 compared to the HG group.

To demonstrate the protective effects of TLS in HG-treated cardiomyocytes, H9c2 cardiomyocytes were incubated with or without different concentrations of TLS, and cell viability and markers of apoptosis and pyroptosis were determined. The MTT assay indicated that HG markedly decreased cell viability, and TLS rescued it (Figure 6(a)). Consistent with the observations in vivo, apoptosis-related proteins, such as the Bax/Bcl-2 ratio, cleaved caspase-3, and pyroptosis-related proteins, including NALP3, caspase-1, IL-18, and IL-1, were overtly increased in H9c2 cardiomyocytes exposed to HG, and TLS treatment alleviated these effects (Figures 6(b)–6(e)).

### 3.7. TLS Activates Autophagy in HG-Treated Cardiomyocytes

**Figure 7:**
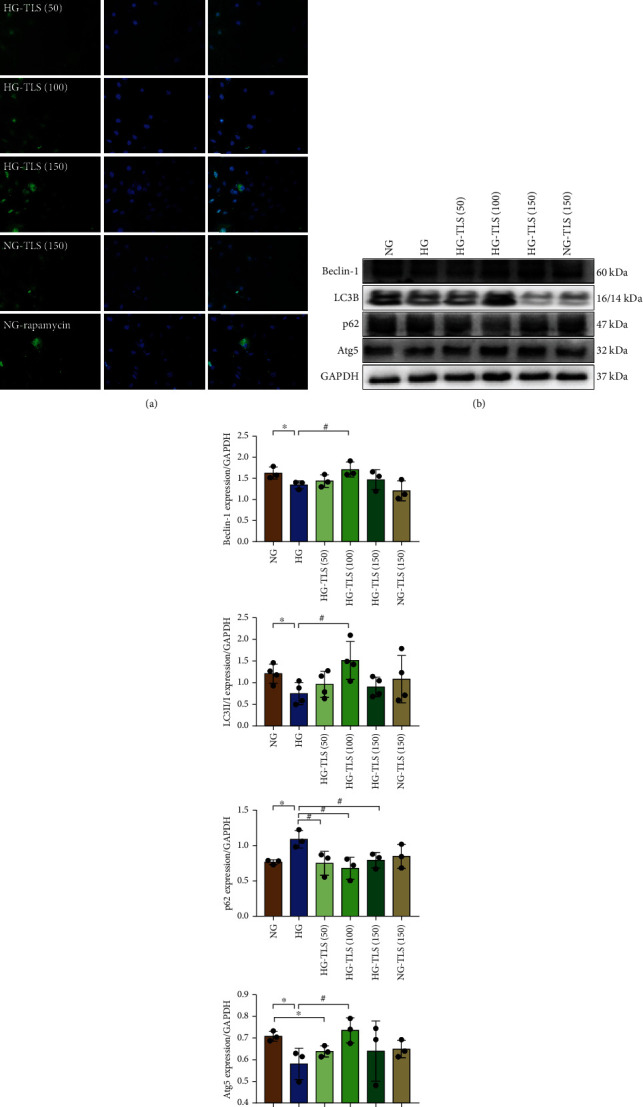
TLS activates autophagy in HG-treated cardiomyocytes. (a) Representative images of GFP-LC3B. (b) Representative protein expression of Beclin-1, LC3B, p62, and Atg5. (c) Quantification of Beclin-1, LC3B, p62, and Atg5 protein expression. GAPDH served as the loading control. Means ± SD, *n* = 3-4 cultures per group. ^∗^*P* < 0.05 compared to the NG group, ^#^*P* < 0.05 compared to the HG group.

Because TLS is an autophagy activator and autophagy participates in the process of DM, autophagic vesicles and autophagy-related proteins were monitored using GFP fluorescence and Western blot, respectively. As shown in Figures 7(a)–7(c), H9c2 cells exhibited a decrease in the number of punctate GFP-LC3 structures after exposure to HG. Beclin-1 and Atg5 expression and the LC3II/LC3I ratio were remarkably downregulated, and p62 was increased in HG-treated cardiomyocytes, the effects of which were attenuated by TLS.

### 3.8. TLS Upregulates the PK2/PKR Signaling Pathway in HG-Treated Cardiomyocytes

**Figure 8:**
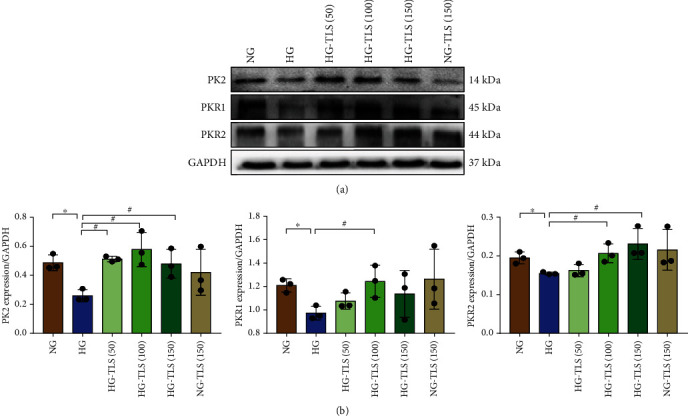
TLS upregulates the PK2/PKR signaling pathway in HG-treated cardiomyocytes. (a) Representative protein expression of PK2, PKR1, and PKR2. (b) Quantification of PK2, PKR1, and PKR2 protein expression. GAPDH served as the loading control. Means ± SD, *n* = 3 cultures per group. ^∗^*P* < 0.05 compared to the NG group, ^#^*P* < 0.05 compared to the HG group.

To verify the role of the PK2/PKR pathway in TLS-induced cardiomyocyte mechanical responses to HG in vitro, Western blot was used to estimate the PK2/PKR signaling pathway. PK2 and PKR were downregulated in H9c2 cardiomyocytes after HG incubation, and TLS treatment abrogated these effects (Figure 8).

### 3.9. A PK2 Antagonist Counteracts the Effects of TLS on Cardiomyocyte Apoptosis and Pyroptosis

**Figure 9:**
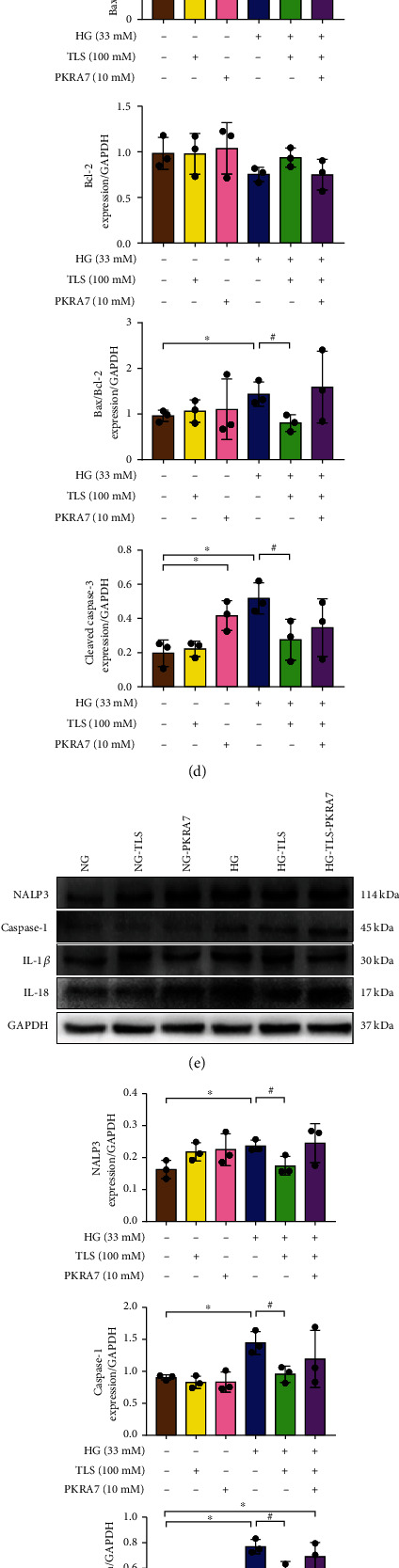
Inhibition of PK2/PKR suppresses the effect of TLS in vitro. (a, b) Representative protein expression and quantification of PK2/PKR. (c, d) Representative protein expression and quantification of apoptosis. (e, f) Representative protein expression and quantification of pyroptosis. (g, h) Representative protein expression and quantification of autophagy. GAPDH served as the loading control. Means ± SD, *n* = 3 cultures per group. ^∗^*P* < 0.05 compared to the NG group, ^#^*P* < 0.05 compared to the HG group, and ^&^*P* < 0.05 compared to the HG-TLS group.

To further clarify whether TLS played a positive role in hyperglycemia-induced cardiomyocytes by activating the PK2/PKR signaling pathway, cardiomyocytes were exposed to HG with or without TLS and the PK2 inhibitor PKRA7. PKRA7 markedly reversed the TLS-induced upregulation of PK2/PKR expression (Figure 9(a)). PKRA7 partially or completely abolished the impacts of TLS on apoptosis- and pyroptosis-related protein expression (Figures 9(c) and 9(e)). PKRA7 failed to exert any effect on the changes in TLS-induced autophagy (Figure 9(g)).

## 4. Discussion

**Figure 10:**
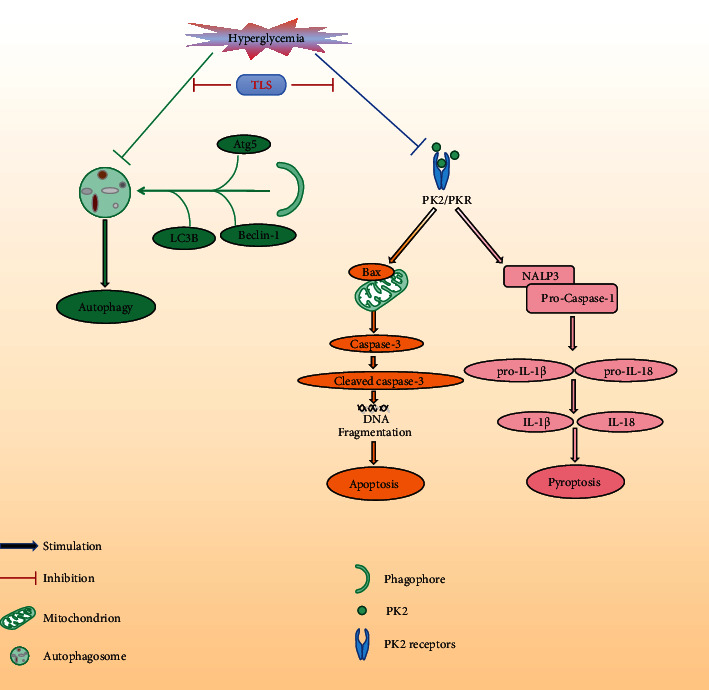
TLS protects against DM-associated heart dysfunction through the PK2/PKR pathway and autophagy.

The remarkable discovery from our present study was that TLS administration alleviated DM-induced cardiac dysfunction, cardiomyocyte apoptosis, and pyroptosis by stimulating the PK2/PKR pathway and increasing autophagy (Figure 10). Although the clinical prevention of DCM remains challenging, our research demonstrated that the PK2/PKR pathway may be the target of TLS in the treatment of DCM.

Glucose is the major impetus for the deterioration of DCM. The present experiment established a diabetic mouse model via the intraperitoneal injection of STZ (50 mg·kg^−1^) for 5 consecutive days. After STZ administration, the blood glucose levels of DM mice were stable and high, which revealed that the diabetic model was established and led to long-term glucose metabolism disorder. TLS slightly improved glucose metabolism, but it did not restore it to normal values, which indicates that the mechanism of TLS is different from conventional anti-diabetic drugs. Therefore, the mechanism of action needs further clarification. Continuous hyperglycemic challenge accelerated the accumulation of TG and TC and impaired cardiac function, which is a consistent pattern with our earlier reports [22]. Echocardiographic assessment quantifies the changes in myocardial function and the process of myocardial remodeling, which has diagnostic and disease progression evaluation significance for the occurrence of DCM [25]. Our study noted pronounced cardiac dysfunction in mice with long-lasting DM, which was partially or overtly reversed by TLS intervention. HE, Masson trichromatic staining, and the myocardial enzyme spectrum supported cardiac injury during DM, and TLS attenuated these effects. Our observations verified the protective effect of TLS on the structure and function of DM mice.

Autophagy is the process of cell self-renewal and removal of damaged organelles [26]. It widely exists in organisms to protect cells from adverse external stimuli and plays an irreplaceable role in the homeostasis of the intracellular environment [27]. Abnormal autophagy (excessive or insufficient) aggravates heart damage, and it is a key step in the pathogenesis of DCM [28]. Atg5, Beclin-1, LC3B, and p62 are markers of autophagy activation in biological tissues. Atg5 and Atg12 form the Atg12-Atg5 conjugate and exert a pivotal role in autophagy [29]. Beclin-1 contributes to invoking autophagy-related proteins to the isolation membrane in the autophagy process [30], and LC3 converts LC3I to autophagosome-bound LC3II, which is involved in the formation and extension of autophagosomes. As a scaffold protein, p62 combines with ubiquitinated substrates to assist in the autophagy process [31]. As an autophagy inducer, TLS induces autophagy via the promotion of LC3 entry into the autophagosome membrane through a mTOR-independent pathway [32, 33]. Our research observed suppressed autophagy in DM-challenged cardiomyocytes, and TLS abolished this inhibition. This result is supported by some experimental findings. (1) Electron microscopy revealed pronouncedly lower levels of autophagic lysosomes in DM mice, and TLS rescued this effect. (2) HG incubation dramatically reduced autophagosome formation (GFP-LC3 puncta) in H9c2 cardiomyocytes, and TLS significantly negated this effect. (3) Western blot analysis indicated downregulation of Atg5, Beclin-1, and the LC3II/LC3I ratio and upregulation of p62 in DM mice and H9c2 cardiomyocytes exposed to HG, and TLS restored these changes.

Downregulation of autophagy triggers the accumulation of broken organelles, which is followed by proapoptotic factors and ROS, and these changes accelerate cardiac dysfunction and the development of DCM [34]. Apoptosis is an orderly gene-regulated process for spontaneous cell death, and various apoptotic stimuli lead to high levels of cardiomyocyte loss and fibrosis, which suggests that suppression of cardiomyocyte apoptosis would restore cardiac function [35]. The proapoptotic protein Bax and the antiapoptotic protein Bcl-2 belong to the B-cell lymphoma/leukemia-2 protein family. A stable Bax/Bcl-2 heterologous dimer exerts an antiapoptotic effect and promotes cell survival and growth [36]. Caspase-3 is a member of the cysteine protease family, and it is the most critical apoptotic execution protease. Activated caspase-3 (cleaved caspase-3) cuts specific substrates that affect DNA replication, transcription, and repair [37]. Consistent with earlier reports [22, 38], long-term HG triggered cardiomyocyte apoptosis in DM mice and increased Bax/Bcl-2 ratio, and cleaved caspase-3 was found in H9c2 cardiomyocytes or mice exposed to HG. TLS inhibited these effects. Therefore, the protective effect of TLS on cardiac injury may be related to the inhibition of cardiomyocyte apoptosis.

New evidence suggests that autophagy regulates a new type of inflammatory cell programmed death, pyroptosis, by rectifying apoptosis [39]. The NALP3 inflammasome is activated under endogenous or exogenous stimulation. The NALP3 inflammasome triggers caspase-1, which mediates cell swelling and the rupture of cell membranes, destruction of cell membrane integrity, and the release of proinflammatory contents in the cytoplasm. NALP3 also mediates stimulation of the inflammatory amplification promoter IL-1*β*, induces the synthesis of the chemokine cytokine IL-18, and leads to the occurrence of intense inflammation. Therefore, the inflammatory factors NALP3, caspase-1, IL-1*β*, and IL-18 are key markers of the pyroptosis process [40]. Data from our study showed that TLS effectively reversed the pyroptosis alterations of NALP3, caspase-1, IL-1*β*, and IL-18 in DM mice and H9c2 cardiomyocytes exposed to HG. These results indicated that restriction of pyroptosis by TLS contributed to the inhibition of programmed cell death.

Recent studies showed that PK2/PKR played an important role in the occurrence and development of cardiovascular diseases [21]. PKR1 gene-knockout mice showed pathological changes, such as cardiac lipid deposition and myocardial systolic and diastolic function damage, which led to ventricular hypoplasia, ventricular septal defects, and embryonic necrosis [41]. The overexpression of PKR1 inhibited apoptosis to protect cardiomyocytes from hypoxia damage [42] and promoted the proliferation of cardiac progenitor cells [20]. PK2 reduced hypoxia/reoxygenation, which induced damage to H9c2 cardiomyocytes via activation of downstream pathways [43]. Previous studies by our research group found that PK2 ameliorated the myocardial cell injury induced by HG and high palmitic acid [44]. Our study showed that activation of the PK2/PKR pathway may be a key mechanism for the cardioprotective role of TLS. This hypothesis was supported by the results. (1) PK2, PKR1, and PKR2 expression was remarkably decreased in diabetic mice hearts and H9c2 cardiomyocytes exposed to HG, and TLS reversed these effects. (2) The PK2 antagonist PKRA7 effectively nullified TLS-induced beneficial responses, such as changes in apoptosis and pyroptosis in the face of HG. These findings support a likely role for the PK2/PKR signaling cascade in the regulation of the TLS response to apoptosis and pyroptosis when faced with glucose toxicity.

In summary, our study confirmed the therapeutic effects of TLS rescue against glucose toxicity-induced myocardial remodeling, fibrosis, cardiac dysfunction, apoptosis, and pyroptosis. The mechanism may be related to the upregulation of autophagy and activation of the PK2/PKR signaling pathway. These results elucidate the role of TLS in DM-induced cardiac abnormalities and lay the foundation for the clinical application of TLS. The results provide a new strategy for the prevention and treatment of DCM.

## Figures and Tables

**Table 1:** Effect of TLS on the organ weight coefficient of diabetic mice ($\bar{x}\pm \text{SD}$, *n* = 10).

Parameter	Control	DM	Control-TLS	DM-TLS
BW(g)	30.48±1.24	17.68±1.88^∗^	30.68±1.82	20.35±3.25^∗^^#^
HW(mg)	150.34±11.38	91.03±7.93^∗^	148.81±10.30	86.16±17.62^∗^
HW/BW(mg/g)	4.94±0.36	5.20±0.70	4.85±0.20	4.23±0.45^∗^^#^
LW(mg)	167.93±12.15	139.84±11.52^∗^	182.73±33.74	148.46±24.05^∗^
LW/BW (mg/g)	5.51±0.33	7.95±0.67^∗^	5.96±1.07	7.41±1.49^∗^

Compared to the control group, ^∗^*P* < 0.05; compared to the DM group, ^#^*P* < 0.05.

## Data Availability

The data used to support the findings of this study are available from the corresponding author upon request.
